# ﻿Interphase nuclei, karyotypes and nuclear DNA amounts in five species of *Oenocarpus* (Arecaceae)

**DOI:** 10.3897/compcytogen.18.117597

**Published:** 2024-05-08

**Authors:** Natália Padilha de Oliveira, Gabriel de Siqueira Gesteira, Maria do Socorro Padilha de Oliveira, Lisete Chamma Davide

**Affiliations:** 1 Departmento de Biologia, Universidade Federal de Lavras, Campus Universitário, Caixa Postal 3037, CEP 37200-000, Lavras, MG, Brazil Universidade Federal de Lavras Lavras Brazil; 2 Embrapa Amazônia Oriental, CPATU, 66095-903, Belém, PA, Brazi Embrapa Amazônia Oriental Belém Brazil

**Keywords:** C-value, cytogenetic analyses, flow cytometry, karyogram, karyotype asymmetry, palms

## Abstract

The genus *Oenocarpus* Martius, 1823 (Arecaceae) includes five species commonly used in Amazonia, especially for their fruits. Little is known about the cytogenetic characteristics and DNA amounts of these species, except for *O.bataua* (Martius, 1823). This study characterized and compared the types of interphase nuclei, the chromosome sets, and estimated the nuclear DNA amounts of *Oenocarpusbacaba* (Martius, 1823), *O.bataua*, *O.distichus* (Martius, 1823), *O.mapora* (H. Karsten, 1857) and *O.minor* (Martius, 1823). Standard cytogenetic analyses and estimates of the nuclear DNA amount by flow cytometry were carried out. These are the first reports of chromosome numbers and DNA amounts, except for *O.bataua*, as is the description of the chromatin distribution in interphase nuclei and karyotype for all species. All species presented 2n = 36, confirming the previous report for *O.bataua*. Differences between karyotype formulas and the positioning of secondary constrictions were observed. There were no significant differences for the nuclear DNA amounts among species. The constancy in chromosome number and variations in karyotype formulas suggest the occurrence of chromosome rearrangement as an important mechanism in *Oenocarpus* speciation.

## ﻿Introduction

The family Arecaceae includes approximately 2,400 species in 190 genera, and is considered to be one of the most abundant among the monocotyledons (Röser 1995; Dransfield et al. 2008). Among the typically tropical genera is *Oenocarpus* Martius, 1823, with nine species (Henderson 1995) found throughout the northern part of South America. Five species have significant economic value for Amazonian communities, especially due to the products derived from their fruits, e.g. used as food, tools and utensils, and for construction (Balick 1986; Henderson 1995; Zambrana et al. 2007). Because of their importance, studies that can increase knowledge of their biology, management and sustainable use of their genetic resources, and their domestication are important.

Cytogenetics offers information for the characterization of germplasm banks, as well as for the management of these resources in genetic breeding programs (Stace 2000). The determination of chromosome number and karyotype are the easiest and cheapest activities among all cytogenetic techniques available, and constitute important information for cytotaxonomic studies (Guerra 2008). There are numerous studies of palm cytogenetics, some including karyotypes, banding patterns and comparison of interphase nuclei morphology, based on chromatin distribution and arrangement (Röser 1993, 1994, 1995, 1999, 2000; Röser et al. 1997; Castilho et al. 2000; Corrêa et al. 2009; Abreu et al. 2011; Battistin et al. 2012; Gaiero et al. 2012; Oliveira et al. 2016; Pereira et al. 2017; Kadam et al. 2023; Witono et al. 2024). However, among the five subfamilies of Arecaceae, the one that has the least cytogenetic information for its species is Arecoideae, which includes *Oenocarpus*, with at least five tribes with no information even on the chromosome number (Dransfield et al. 2008). For *Oenocarpus* only *O.bataua* (Martius, 1823) has a chromosome number report: 2n = 36 (Röser et al. 1997).

Studies involving species of this subfamily can contribute to understanding karyotype evolution in the Arecaceae.

The analysis of nuclear DNA amounts by flow cytometry in plant species allows estimation of genome sizes, for comparison with chromosome numbers, ploidy levels and detection of numerical alterations (Bennett and Leitch 1995; Doležel and Bartos 2005). The amount of information for palms has increased recently (Rival et al. 1997; Sandoval et al. 2003; Madon et al. 2008; Abreu et al. 2011; Cepeda-Cornejo et al. 2012; Farias Neto et al. 2016; Jatt et al. 2019; Sharma et al. 2023). However, the most extensive study, which included 83 species in all five subfamilies (Röser et al. 1997), used the microdensitometry methodology of Feulgen (Teoh and Rees 1976).

In this context, this study characterized and compared interphase nuclei morphology and chromosome sets, and estimated the amount of nuclear DNA for *O.bacaba* (Martius, 1823), *O.bataua*, *O.distichus* (Martius, 1823), *O.mapora* (H. Karsten, 1857) and *O.minor* (Martius, 1823). These are the five most useful species, and samples are maintained for study and improvement by Embrapa Eastern Amazon, Amazon, Belém, Pará.

## ﻿Material and methods

### ﻿Plant material

Seeds obtained from three accessions of *O.bacaba*, *O.bataua*, *O.distichus*, *O.mapora*, and *O.minor*, kept at the Active Germplasm Bank of Embrapa Eastern Amazon, in Belém, Pará, Brazil, were used in both analyses. Analysis was authorized by the federal institutions **CGEN** (process no. 02000.002611/2012-60) and **IBAMA** (process no. 02001.001558/2006-21). Vouchers are deposited in the **IAN** Herbarium, Belém, and details of each accession are presented in Table 1. After mechanical processing, seeds were set to germinate in BOD at 28 °C with a 12 h photoperiod. Seedlings obtained from each species were kept in a greenhouse at the Federal University of Lavras **UFLA**, Lavras, Minas Gerais, Brazil.

**Table 1.** Number of individuals and origin of *Oenocarpus* sp. genotypes used on analyses.

Species	Number of indivuduals	Origin
*O.bacaba*	1	Magazão-AP
	1	Macapá-AP
	1	Porto Grande-AP
*O.bataua*	1	Irituia-PA
	2	Anajás-PA
*O.mapora*	3	Abaetetuba-PA
*O.distichus*	3	Oriximiná-PA
*O.minor*	3	Terra Santa-PA

### ﻿Cytogenetic analysis

Root tips were pre-treated with colchicine 0.1% for 5 h at 4 °C, fixed in Carnoy’s solution (3:1 alcohol/acetic acid) and stored at -20 °C. Slide preparation used the squashing technique (Guerra and Souza 2002) following cell wall digestion with cellulase/pectinase (100U/200U) for 2 h at 37 °C. Aceto-orcein 1% was used to stain the samples for the analysis of mitotic metaphases, while 10% Giemsa (diluted in phosphate buffer, pH 6.8, following Guerra and Souza 2002) was used to analyze interphase nuclei.

The slides were examined in a bright-field microscope (Leica DMLS), equipped with a digital camera (Nikon Digital Sight DS-Fi1) to digitalize the best nuclei and metaphases. In order to evaluate chromatin organization at interphase, 500 nuclei were analyzed for each species. Ten metaphases were selected to determine the chromosome number for each species, of which five were used for karyotype construction, after obtaining the measurements of the short (s) and long (l) arms of the chromosomes, using the IMAGE TOOL 3.00 program from The University of Texas Health Science Center in San Antonio (http://ddsdx.uthscsa.edu/dig/download.html). The total length of the chromosome (**Cti** = l + s), arm ratio (**AR** = l/s), total length of the haploid set (**TLHS** = ΣCti/2), and relative length of each chromosome (**RL** = Cti/TLHS × 100), and were estimated. Chromosome morphology was described based on arm ratios, following Levan et al. (1964). Karyograms were obtained using Adobe Photoshop CS2. To compare the mean sizes of the chromosome sets among species, an analysis of variance of a completely randomized design was used and means were compared with the Tukey test at 5%, using the R package in R (R Development Core Team 2011).

For karyotype asymmetry, the intrachromosomal asymmetry (A1), which quantify the relative differences in the centromere position among chromosomes of a complement, and the interchromosomal asymmetry (A2), which quantify the heterogeneity in chromosome size, were calculated following Zarco (1986). Karyotype asymmetry was also calculated following Stebbins (1971), which proposes a classification based on three degrees of difference between the largest and the smallest chromosome of the complement, combined with four degrees regarding the proportion of chromosomes which are acro- or telocentric.

### ﻿Estimates of nuclear DNA amounts

Nuclear DNA amounts were estimated by flow cytometry, using leaf tissue, following Galbraith et al. (1983). Propidium iodide (1 mg/ml) was used as a fluorochrome and for internal standard, a pretest was conducted, after which *Viciafaba* (Linnaeus, 1753) (2C = 26.9 pg) was chosen because of the quality of graphics obtained. For each species three specimens, the same accessions used in the cytogenetic analysis, were analyzed and three estimates were made for each one of them. The analyses were carried out in a FacsCalibur cytometer (BD Biosciences, San Jose, CA, USA), the histograms were obtained using the software Cell Quest (Becton Dickinson and Company, San Jose, CA, USA) and analyzed with the software WinMDI 2.8. Nuclear DNA amounts (2C) of each accession were estimated as (sample G1 peak mean/ standard G1 peak mean) × standard 2C value. To compare the mean nuclear DNA amounts among species, an analysis of variance of a completely randomized design was used and means were compared with the Tukey test at 5%, using the R package in R (R Development Core Team 2011).

## ﻿Results

**Figure 1.**
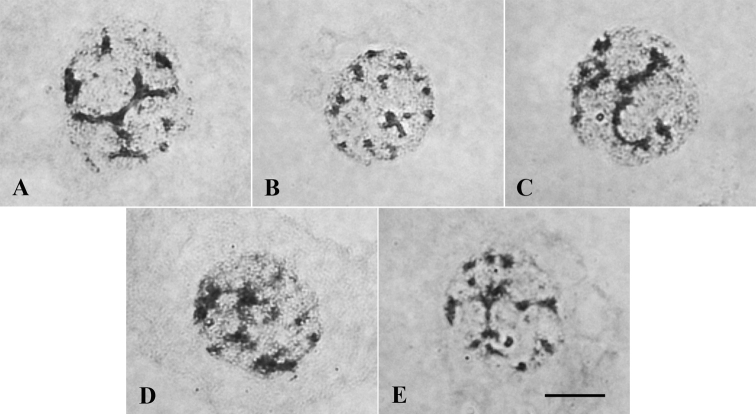
Semi-reticulate interphase nuclei found for *Oenocarpus* spp. **A***O.bacaba***B***O.bataua***C***O.distichus***D***O.mapora***E***O.minor*. Scale bar: 10 µm.

Only semi-reticulate interphase nuclei were found (Fig. 1A–E), which are characterized by the presence of strongly pigmented chromatin structures with irregular edges, known as chromocenters (Guerra 1987).

**Figure 2.**
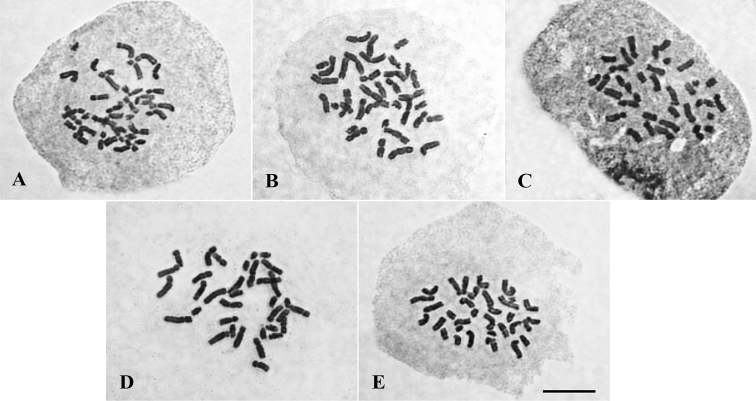
Mitotic metaphases of *Oenocarpus* spp. showing 2n = 36 **A***O.bacaba***B***O.bataua***C***O.distichus***D***O.mapora***E***O.minor*. Scale bar: 10 µm.

**Figure 3.**
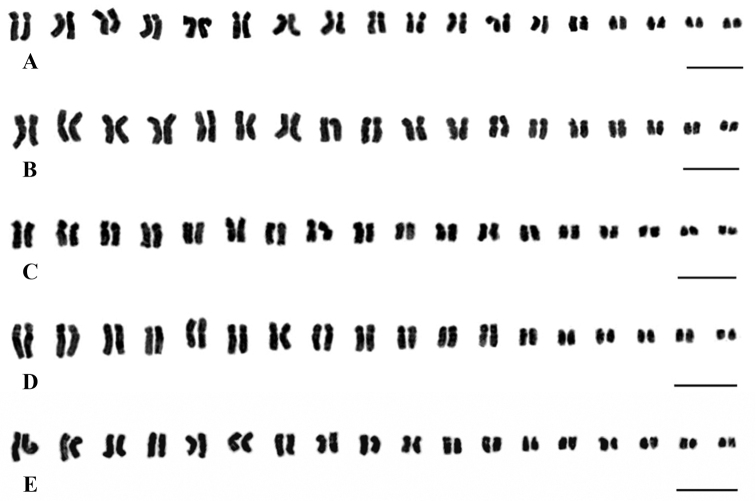
Karyograms of *Oenocarpus* spp. based on the metaphases displayed previously **A***O.bacaba***B***O.bataua***C***O.distichus***D***O.mapora***E***O.minor*. Scale bar: 10 µm.

**Figure 4.**
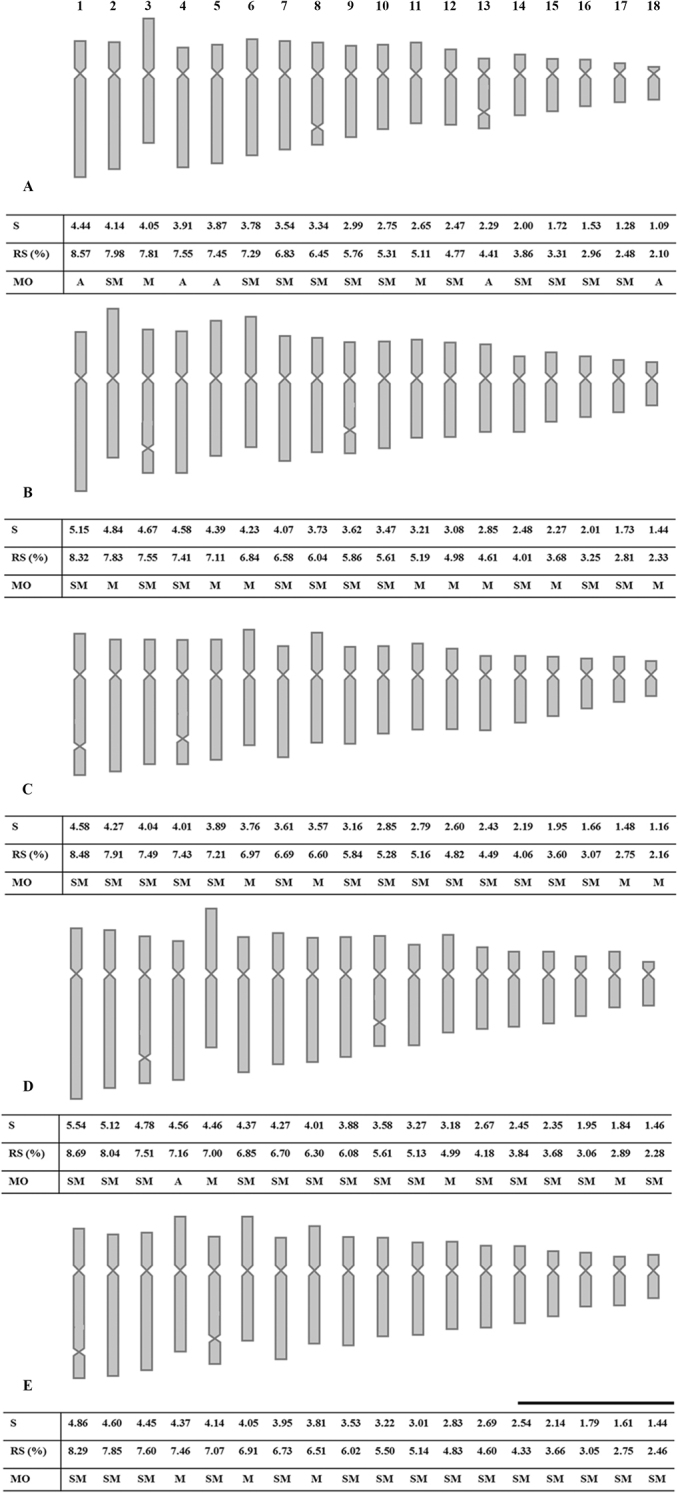
Idiograms of *Oenocarpus* spp. including length (L), relative length (RL), and morphology (MO) of each chromosome pair **A***O.bacaba***B***O.bataua***C***O.distichus***D***O.mapora***E***O.minor*. Scale bar: 5 µm.

The chromosome number was also constant among species: 2n = 36 (Fig. 2A–E). However, there was variation in size, morphology and position of secondary constrictions (Figs 3A–E, 4A–E). The karyotype formulas found for the species were the following: *O.bacaba* (2M + 11SM + 5A), *O.bataua* (8M + 10SM), *O.distichus* (4M + 14SM), *O.mapora* (3M + 14SM + 1A) and *O.minor* (3M + 15SM). Total length for the haploid set was higher for *O.mapora*, with 63.7 μm, while *O.bacaba* showed the lowest value, 51.8 μm. However, the analysis of variance did not detect differences among mean values (Table 2).

**Table 2.** Mean values of total length of haploid set and DNA amount of *Oenocarpus* sp.

Species	*O.bacaba*	*O.bataua*	*O.distichus*	*O.mapora*	*O.minor*
TLHS (µm)	51.835a	61.823a	54.001a	63.712a	59.053a
2C DNA amount (pg)	6.794a	6.457a	6.554a	6.483a	6.960a

Same letter indicates group formed by Tukey test at 5%.

In the karyotypes of the five species two chromosome pairs with secondary constrictions were observed, all located in the terminal portion of the long arm. In *O.bacaba* secondary constrictions occurred in chromosome pairs 8 and 13, and presented 0.60 and 0.53 µm, respectively (Fig. 4A); in *O.bataua* they occurred in pairs 3 and 9, with 0.81 and 0.79 µm, respectively (Fig. 4B); in *O.distichus* in pairs 1 and 4, with 0.95 and 0.82 µm, respectively (Fig. 4C); in *O.mapora* in pairs 3 and 10, with 0.86 and 0.80 µm, respectively (Fig. 4D); and in *O.minor* in pairs 1 and 5, with 0.88 and 0.85 µm, respectively (Fig. 4E).

**Figure 5.**
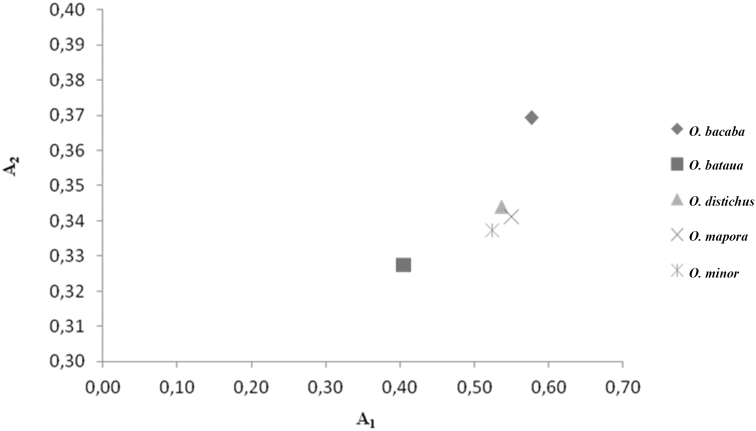
Scatterplot for karyotype asymmetry of the five *Oenocarpus* species based on the intrachromosomal asymmetry index (A1) and the interchromosomal asymmetry index (A2), according to Zarco (1986).

The results of the karyotype asymmetry analysis were coincident for the methodologies proposed by Stebbins (1971) and Zarco (1986). The greater symmetry as presented by *O.bataua*, classified in the category 2b (Stebbins 1971), as well as a lower intrachromosomal asymmetry (A1) and a lower variation in size between the chromosomes (A2) (Zarco 1986). The species *O.distichus*, *O.mapora* and *O.minor* were grouped in the same category, 3b (Table 3). In Fig. 5 it is noted that *O.distichus*, *O.mapora* and *O.minor* formed a similar group. The species *O.bacaba* presented higher values for A1 and A2 and was classified in the 3c category, thus representing the most asymmetrical of the five species in both methodologies (Table 3).

**Table 3.** Karyotype asymmetry for the five *Oenocarpus* species according to Stebbins (1971) and Zarco (1986).

Species	Karyotype Asymmetry		
	Stebbins	Zarco (A1 e A2)	
*O.bacaba*	3c	0.5767	0.3693
*O.bataua*	2b	0.4046	0.3274
*O.distichus*	3b	0.5361	0.344
*O.mapora*	3b	0.5497	0.3412
*O.minor*	3b	0.5241	0.3373

As for the 2C amount of nuclear DNA, the average values found for the species varied between 6.46 pg, in the *O.bataua*, and 6.96 pg, in the *O.minor* (Table 2). The analysis of variance did not detect differences among averages values.

## ﻿Discussion

Except for *O.bataua*, the chromosome counts obtained in this study, as well as the karyotypes, the morphology of interphase nuclei and nuclear DNA amounts of the species are new. The chromosome number found for the *O.bataua* confirms the prior report (Röser et al. 1997), but the 2C DNA value differs by more than 1 pg from that presented by those authors. It is important to point out that the methodology used by those authors, Feulgen’s microdensitometry, estimates the amount of DNA in a different way than flow cytometry used in this study (Röser et al. 1997). In the literature, although correlated results for DNA amounts using both techniques are frequent (e.g. Baranyi and Greilhuber 1996), differences in DNA amounts for the same species when estimated with both techniques have been reported, although there is no agreement as to the explanation for this fact (Schifino-Wittmann 2001). The analysis of the specimens studied by Röser et al. (1997) using flow cytometry would help to verify whether the difference found in this study is due to the methodology or whether there is intraspecific variation, as found in *Cocosnucifera* (Linnaeus, 1753) by Gunn et al. (2015). Abreu et al. (2011) also found a different 2C value than found by Röser et al. (1997) for *Acrocomiaaculeata* (Loddiges ex. Martius, 1823), and the authors suggested that the different methodologies and different origins of the genotypes probably influence the estimation of nuclear DNA amounts.

As for the type of interphase nuclei, in the Arecaceae there are reports on the occurrence of three types of nuclei, reticular, semi-reticular and areticular, and this characteristic has proven to be constant among congener species, and sometimes even in superior taxonomic levels such as tribes (Röser 1994). Our results for these five *Oenocarpus* species confirm the pattern. According to Guerra (2000), the semi-reticular type of nucleus is typical of species with medium sized chromosomes, e.g., 3 to 5 µm, as found in this study.

The number of chromosomes found for the *Oenocarpus* species was the same as that found for other species of the tribe Euterpeae (Battistin et al. 2012; Oliveira et al. 2016). The constancy in the number of chromosomes among closely related species is quite common among groups of Arecaceae (Röser 1994, 1995; Dransfield et al. 2008; Corrêa et al. 2009).

The chromosome number for Arecaceae species varies from 2n = 26 to 2n = 36 (Röser 1999; Dransfield et al. 2008). The number 2n = 36 is the most commonly found in some subfamilies, such as the Coryphoideae (Röser 1994; Dransfield et al. 2008), in which almost all the species present this number. According to the same authors, the subfamily Arecoideae, in turn, is the most diversified, with 2n = 32 chromosomes being the most common number, although the number 2n = 36 is also quite expressive in tribes such as Euterpeae.

Other chromosome numbers have been reported for species of the subfamily Arecoideae, to which the genus *Oenocarpus* belongs. Castilho et al. (2000), Corrêa et al. (2009), Battistin et al. (2012), and Pereira et al. (2017) found 2n = 32 chromosomes for *Elaeisguineensis* (Jacquin, 1763), five species of *Butia* (Beccari, 1916), *Archontophoenixalexandrae* ((F. Muell.) H. Wendland et Drude, 1875), and *Cocosnucifera*, respectively; Abreu et al. (2011) reported the number 2n = 30 for the *Acrocomiaaculeata* species; Cepeda-Cornejo et al. (2012) found a variation for different species of *Chamaedorea* (Willdenow, 1806), 2n = 32 for *C.tepejilote* (Liebmann, 1849) and *C.alternans* (Willdenow, 1880), and 2n = 26 for *C.pinnatifrons* (Oersted, 1858) and *C.ernesti-augusti* (Wendland, 1852).

As for the secondary constrictions, in Arecaceae species it is common to find one or two pairs of chromosomes bearing nucleolus organizer regions (NORs), but five pairs occur in *Pseudophoenixvinifera* ((Mart.) Beccari, 1912) (Röser 1994). Those regions have been found more frequently at the end of the short arm of the chromosomes (Röser 1999; Castilho et al. 2000; Pereira et al. 2017). Roa and Guerra (2012) pointed out a tendency for the quantity and location of 45S rDNA sites for angiosperm species in general to be similar to that found palms (Röser 1999). Although the secondary constrictions verified in *Oenocarpus* were found at the end portion of the chromosomes, they were all detected on the long arm. Oliveira et al. (2016) also found secondary constrictions on the long arm for *E.edulis* (Martius, 1824) and *E.precatoria* (Martius, 1842). It is important to stress that the subfamily Arecoideae has the least cytogenetic information. Therefore, it needs to be verified whether this difference in positioning found for the secondary constrictions is exclusive to *Oenocarpus*, or whether it is a characteristic shared by other genera of this subfamily.

Based on the karyotypes of the species studied here and emphasizing the differences between the positioning of the centromere and the secondary constrictions, it can be inferred that alterations, especially structural rearrangements, such as translocations and pericentric inversions, as well as activities related to the transposable elements, accumulated during the evolution of this genus. According to Stebbins (1971), such rearrangements are important in evolution, as they increase karyotype asymmetry and the differentiation among chromosome sets.

Regarding the nuclear DNA amounts, similar results have been found for other palm species from the same subfamily. *Cocosnucifera*, *Elaeisguineensis* and *Attalea* spp. (Kunth, 1816), all with 2n = 32 chromosomes, and *Acrocomiaaculeata* (2n = 30) had their 2C DNA value estimated at 3.76 pg (Sandoval et al. 2003), 3.86 pg (Rival et al. 1997), 3.80 pg (NP Oliveira, unpubl. res.), and 5.81 pg (Abreu et al. 2011), respectively. Nevertheless, much higher 2C values have been found for species from different subfamilies of Arecaceae, *e.g.*, *Iriarteadeltoidea* (Ruiz et Pavón, 1798), *Pinangacoronata* (Blume, 1839), and *P.subintegra* (Ridley, 1907), all from the same subfamily Arecoideae to which *Oenocarpus* belongs and with chromosome number 2n = 32, but with 2C values estimated at 24.56, 17.71, and 27.81 pg, respectively; *Trithrinaxcampestris* (Drude et Griseback, 1879) (2n = 36), and *Caryotaurens* (Linnaeus, 1753) (2n = 34), from Coryphoideae, with 17.15 ± 0.07 pg (Gaiero et al. 2012) and 13.22 pg (Röser et al. 1997), respectively.

The nuclear DNA amount in palm species, unlike the number of chromosomes, presents large variation (Röser et al. 1997). Differences of more than 14 times between the smallest and the largest genome size were found, considering only diploid palm species from different genera and subfamilies, which explains the observed variation found for chromosome sizes in the same species (Röser 1994, 2000; Röser et al. 1997). Despite the remarkable diversity found in this family, nuclear DNA amounts seldom vary much within genera, as found here for these five *Oenocarpus* species, and even at higher taxonomic levels (Röser et al. 1997; Röser 2000). Furthermore, the amount of DNA in Arecaceae species seems to follow the same trend as chromosome number, that is, reduction (Röser 2000), but does not seem to be proportional to the chromosome number reduction. Castilho et al. (2000) suggested the amplification of dispersed repetitive DNA sequences as one of the mechanisms responsible for such variation in nuclear DNA amounts. Nevertheless, there is still a lot of research to be done to better understand this diversity.

The five species of *Oenocarpus* follow the majority of the tendencies identified in the Arecaceae family, such as the constancy in chromosome number within the genus and little variation for nuclear DNA amounts. However, other studies are seeking to understand more clearly the mechanisms involved in the karyotype differentiation of these species, as well as consolidating phylogenetic inferences suggested for this genus.

## Supplementary materials

Supplementary material 1 Size of each chromosome and total length of haploid set (TLHS) for *Oenocarpus* species Data type xlsx Explanation note Asterisk (*) indicates second constriction. https://binary.pensoft.net/file/1043758This dataset is made available under the Open Database License (http://opendatacommons.org/licenses/odbl/1.0/). The Open Database License (ODbL) is a license agreement intended to allow users to freely share, modify, and use this Dataset while maintaining this same freedom for others, provided that the original source and author(s) are credited.Natália Padilha de Oliveira, Gabriel de Siqueira Gesteira, Maria do Socorro Padilha de Oliveira, Lisete Chamma Davide
